# Pseudotumour: an uncommon complication of severe haemophilia

**DOI:** 10.1093/bjrcr/uaae019

**Published:** 2024-06-20

**Authors:** Alex Kiu, Isaac Yang, Tiffany Fung, Rehana Jaffer, Marie-Helen Martin

**Affiliations:** Department of Diagnostic Radiology, McGill University Health Center, Montreal, QC H4A 3J1, Canada; Department of Diagnostic Radiology, McGill University Health Center, Montreal, QC H4A 3J1, Canada; Department of Diagnostic Radiology, McGill University Health Center, Montreal, QC H4A 3J1, Canada; Department of Diagnostic Radiology, McGill University Health Center, Montreal, QC H4A 3J1, Canada; Department of Diagnostic Radiology, McGill University Health Center, Montreal, QC H4A 3J1, Canada

**Keywords:** Pseudotumour, haemophilia, musculoskeletal

## Abstract

Pseudotumours are uncommon complications of haemophilia, occurring in 1%-2% of patients with haemophilia.^1^^,^^2^ It is a slowly expanding haematoma as a result of recurrent haemorrhage, surrounded by a fibrous capsule. It can occur in both bone and soft tissue, and progressive enlargement may result in bone destruction and/or muscle and skin necrosis. Pseudotumours by themselves are usually painless though its mass effect can result in nerve compression resulting in pain or neurologic symptoms. It may also predispose to pathologic fractures (as in our case) and superimposed infections.^2^^,^^3^

## Clinical presentation

A 72-year-old man with a history of haemophilia presented to our emergency department with atraumatic right knee pain for 2 days. The pain is exacerbated by weight bearing. Physical exam demonstrated reduced range of motion, but no swelling or overlying skin changes. Laboratory values are as follows: haemoglobin 111 g/L, white blood cell 12.1 × 10^9^/L, platelets 221 × 10^9^/L, INR 0.98, PT 13.9 s, PTT 73.8 s.

## Imaging findings

**Figure 1.**
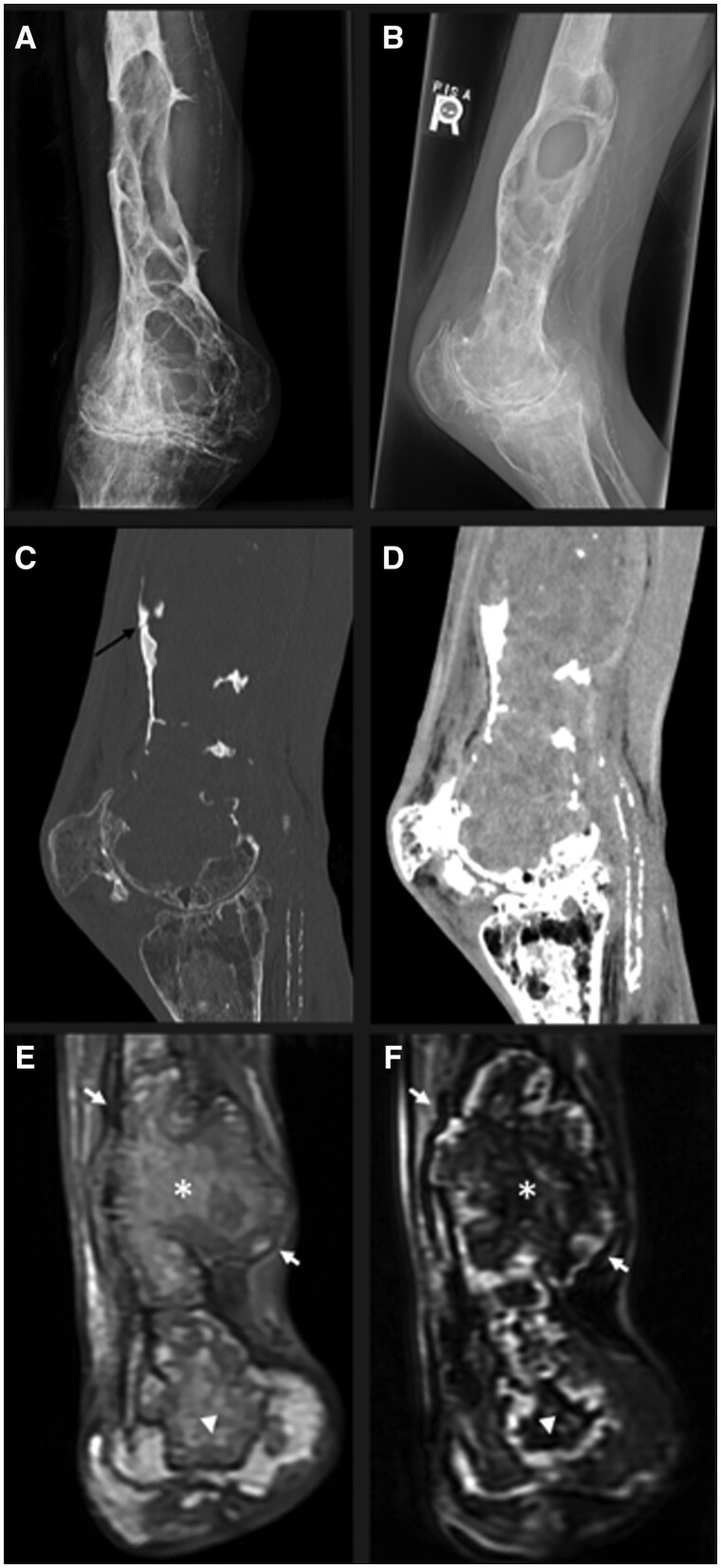
(A) Frontal and (B) lateral radiographs of the knee shows a large lobulated lytic lesion with well-defined margins in the distal femur. Also seen is severe tricompartmental knee arthropathy representing haemophiliac arthropathy. CT in (C) bone and (D) soft-tissue windows shows the extent of osseous destruction secondary to the large soft tissue mass, and the pathologic fracture that was radiographically occult (black arrow). (E) T1 fat-saturation and (F) T2 Short-Tau Inversion-Recovery MRI images in coronal planes showing the mass to be of heterogeneous signal, with regions of high signal intensity on T1-weighted image, and low signal intensity on T2-weighted image representing intracellular methaemoglobin (*), and regions of low T1, high T2 signal intensity representing extracellular methaemoglobin (arrow head). The mass is encapsulated by a well-defined hypointense fibrous capsule (white arrows).

A radiograph was performed, which revealed a large lobulated expansile lytic mass with well-circumscribed borders within the distal femur with large regions of bone destruction. A subsequent CT showed medullary expansion secondary to a large heterogeneous soft tissue mass and a subtle non-displaced fracture (Figure 1). An MRI was performed, which demonstrated a mass of heterogeneous T1 and T2 signals with a well-defined peripheral hypointense rim (Figure 1). Based on the appearance, the diagnosis of haemophilia-associated pseudotumour was proposed.

Radiographically, osseous pseudotumour was present as well-margined, expansile lytic masses. CT and MRI are useful in determining the extent of the pseudotumour and the anatomic relationship between the pseudotumour and adjacent structures. CT is useful in the evaluation of bones, especially for the detection of occult fractures. MRI is superior for assessment of soft tissues and intramedullary spaces. MRI appearance will vary depending on the age of haemorrhagic products, but will invariably depict a low signal rim representing the fibrous capsule and hemosiderin, which in turn confirms remote blood products.^2^^,^^3^ MRI can also detect acute intralesional haemorrhage, with acute haemorrhage appearing isointense on T1-weighted images and hypointense on T2-weighted images.^3^

The diagnosis of pseudotumour can be made confidently on the basis of imaging findings in a patient with severe coagulation disorder. This is of critical importance as biopsy or surgical drainage of the pseudotumour has a high risk for complications, including life-threatening bleeding, fistula formation, and infection, and generally contraindicated.^3^^,^^4^

## Differential diagnosis

The differential diagnosis would include aggressive lesions or processes such as infection, amyloid arthropathy or Ewing’s sarcoma in a younger population. The intraosseous lytic process can mimic tumour-like lesions such as aneurysmal bone cysts or brown tumors.^5^ Metastases would also be in the differential diagnoses with primary consideration being metastases yielding lytic lesions such as from renal cell, melanoma, or thyroid cancer. However, if this presentation was complimented with a history of coagulation disorder, haemophilic pseudotumour would be the principal diagnosis.

## Treatment and follow-up

**Figure 2.**
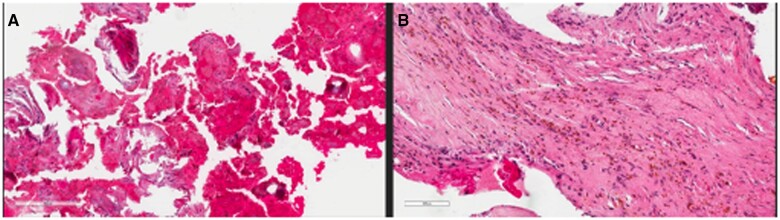
Microscopy of biopsy sample (haematoxylin and eosin stain) showing: (A) Multiple fragments of fibrous tissue intermixed with blood clot. (B) Numerous brown-stained hemosiderin deposits are seen in the fibrous tissue, indicating remote bleeding.

A biopsy was performed under ultrasound guidance, which confirmed the radiologic diagnosis (Figure 2). The patient was immobilized in a Zimmer splint and treated with recombinant factor VIII prior to being discharged.

## Discussion

Haemophilia is an inherited disorder leading to repetitive bleeds secondary to coagulation abnormality.^1^

While uncommon, haemophilic pseudotumours are an important complication to consider in patients with known haemophilia. The recurrent haemorrhage results in a chronic, indolent but expansile and aggressive process that can lead to pathological fractures.^5^ Haemophilic pseudotumours are predominantly either osseous or soft tissue but often transgress anatomic borders, resulting in a mixed appearance.^5^

The intraosseous pseudotumours result from recurrent haemorrhage into the bone, involving most frequently the femur, pelvis, tibia, and hands.^5^ These lesions typically present as well-defined, multi-loculated expansile lytic lesions involving the metadiaphysis and occasionally extending into the epiphysis.^5^ There are often dystrophic calcifications, endosteal scalloping with cortical thinning, or thickening with peripheral sclerosis.^5^ Notably, these destructive changes can lead to pathological fracture, such as in this case.^1^^,^^5^

Repetitive bleeding into the joint and soft tissues pseudotumors is common, with associated destruction of the joint. Joint contractures and soft tissue pseudotumors result from the fibrous tissue proliferation secondary to haemorrhage.^5^ This process can occur both within the muscle or extramuscular compartments. More rarely, these chronic pseudotumors can become infected as a secondary complication.^6^ The soft tissue form can be present in the absence of bony changes, making careful evaluation of the soft tissues crucial in the diagnosis.^5^

In children, the inflammatory response in the synovium can affect the epiphysis, causing growth deformities and subsequent leg-length discrepencies.^1^ Classic imaging findings also include squaring of the patella and widened intercondylar notch.^1^

Management of acute bleeds with medical treatment along with rest and physiotherapy is first line for haemophilic arthropathy/pseudotumors. With progression, management may need to be escalated to low-dose radiation therapy, curettage, or even surgical resection with bonegraft.^7^^,^^8^

## Learning points

- Pseudotumours are uncommon complications of haemophilia.
- Pseudotumours as it name suggests may mimic aggressive tumours clinically and particularly in imaging.
- Haemophilic pseudotumours may have non-specific signal characteristics on T1- and T2-weighted imaging depending on the stage of evolution of haemorrhagic products. However, they are characteristically well circumscribed by a hypointense peripheral fibrous capsule.
- Understanding and appreciating that haemophilic pseudotumours can be diagnosed with imaging may prevent unnecessary interventional/surgical biopsy, alongside the frequently associated high risk for severe complications.
